# Flooding and Soil Properties Control Plant Intra- and Interspecific Interactions in Salt Marshes

**DOI:** 10.3390/plants11151940

**Published:** 2022-07-26

**Authors:** Elisa Pellegrini, Guido Incerti, Ole Pedersen, Natasha Moro, Alessandro Foscari, Valentino Casolo, Marco Contin, Francesco Boscutti

**Affiliations:** 1 Department of Agricultural, Food, Environmental and Animal Sciences, University of Udine, Via delle Scienze 206, 33100 Udine, Italy; guido.incerti@uniud.it (G.I.); natasha.moro@hotmail.it (N.M.); alessandro.foscari@uniud.it (A.F.); valentino.casolo@uniud.it (V.C.); marco.contin@uniud.it (M.C.); francesco.boscutti@uniud.it (F.B.); 2 Department of Biology, University of Copenhagen, Universitetsparken 4, 2100 Copenhagen, Denmark; opedersen@bio.ku.dk

**Keywords:** biotic stress, edaphic stressors, hydroperiod, plant-plant interaction, plant traits, saltmarsh, SGH, wetlands

## Abstract

The stress gradient hypothesis (SGH) states that plant-plant interactions shift from competition to facilitation in increasing stress conditions. In salt marshes, edaphic properties can weaken the application of the SGH by amplifying the intensity of flooding and controlling plant zonation. We identified facilitative and competitive interactions along flooding gradients and tested the role of edaphic properties in exacerbating stress and shaping plant-plant interactions. Morphological traits of two target halophytes (*Limonium narbonense* and *Sarcocornia fruticosa*), flooding intensity, soil texture and soil organic C were recorded. The relative plant fitness index was assessed for the two species based on the relative growth in plurispecific rather than monospecific plant communities. Plant fitness increased with increasing stress supporting the SGH. *L. narbonense* showed larger fitness in plurispecific stands whereas *S. fruticosa* performed better in conspecific stands. Significant intra- or interspecific interactions were observed along the stress gradient defined by the combination of flooding and clay content in soil. When considering the limited soil organic C as stressor, soil properties were more important than flooding in defining plant-plant interactions. We highlight the need for future improvements of the SGH approach by including edaphic stressors in the model and their possible interactions with the main abiotic drivers of zonation.

## 1. Introduction

Wetlands are considered model habitats for the study of plant acclimation traits and plant-plant interactions in response to abiotic stresses because of their low species richness [1] and remarkable stress responses [2]. Soil anoxia and elevation explain most of the variation in plant acclimation and zonation [3]. Soil anoxia caused by flooding [4] results from a critical lack of oxygen supply to plant roots, due to the dramatic reduction of gas diffusion in water [5]. Elevation determines the frequency and magnitude of flooding, triggering soil anoxia processes. Moreover, plants growing at low elevation are prone to partial or complete submergence, which results in a decline in photosynthetic rates due to the lower CO_2_ diffusion and light penetration in water compared to air [6]. If submergence occurs during the night, the slow diffusion can restrict O_2_ supply to the shoot resulting in O_2_ shortage in the belowground tissues [7]. Glycophytes do not tolerate such intense stresses, whereas flood-tolerant species and halophytes can cope with both tissue anoxia and submergence. Flooding often interplays with biotic interactions, consequently affecting growth and fitness of flood-tolerant species. In this light, plant-plant interactions actively contribute to the final structure of the community and their changes along flooding gradients [8,9]. Therefore, plant distribution does not only rely on species tolerance to flooding, but it is also highly dependent on biotic interactions where a species can limit or promote the establishment and growth of other individuals.

Both interspecific competition and facilitation occur in salt marshes and the net balance of these interactions changes in magnitude and direction along the flooding–stress gradient. This concept is the core of the stress gradient hypothesis (SGH) [10]. The SGH states that net interspecific interactions shift from competition to facilitation at increasing stress, with a severity-interaction relationship showing a monotone increase along the stress gradient [11]. The response in terms of plant fitness, predicted by the SGH, is the net result of the reducing competition and increasing facilitation at progressively higher stress levels. In salt marshes, positive interactions are more likely to occur at low elevated areas, as response to the reduction in plant-plant competition under harsh environmental conditions. Here, facilitation results mainly in the direct or indirect mitigation of salinity [12], flooding [13,14] or nutrient-limitation [15] stresses.

The application of the SGH has led to criticisms and needs further improvements to account for, e.g., species-specific tolerance to a stress and for the dichotomy of resource vs. non-resource stress type [16]. Indeed, some studies showed that facilitative interactions could prevail at intermediate stress levels, while the loss of fitness of the nursing species (i.e., the species facilitating other, heterospecific beneficiaries) at harsher stress intensity could lead to higher competition, resulting in a hump-shaped severity-interaction relationship [17]. However, the network of interactions is complex: it can be asymmetrical and differs according to the trait used to assess plant performance [18] and facilitation. Moreover, competition can occur not only between species but also among individuals in monospecific stands, with a dynamic behaviour of plant performance emerging from the interplay of changing stress levels and plant ontogeny [19]. After the pivotal theoretical paper by Bertness and Callaway [10], largely based on evidence from wetlands, most SGH observations have been reported from alpine ecosystems [17,20] with elevation used as a gradient of increasing cold or soil erosion stresses, or from grasslands with herbivory pressure considered as the main stressor [21]. Although the SGH was confuted in some of these studies [22,23], its core idea remains convincing in the salt marsh context, especially at the local scale [24]. In this context, it is of foremost relevance to test whether a monotone or hump-shaped severity-interaction relationship also occurs in habitats where empirical observations led to the formulation of the SGH. Consequently, this study aimed to assess whether the SGH still holds for the typical stressors of wetland systems, with salt marsh plant communities conceptually replacing mountain or grassland ecosystems and flooding intensity acting as the main limiting factor in place of altitude or herbivory pressure.

Importantly, application of the SGH should consider that one stressor rarely acts alone. Salt marsh soils are affected by floods but also by salts present in the brackish tidal waters or, at low elevation, flooding stress can combine with soil erosion. The SGH therefore needs to include multiple gradients for which each stressor relies on different responses by each species [25]. In salt marshes, soil texture is a main determinant of soil porosity, which can exacerbate the effects of flooding by delaying water drainage. Soil texture affects the biogeochemistry and microbial activity of flooded soils [26] and, together with the presence of sulfidic materials, contributes to plant zonation [27]. Huckle et al. [28] reported differences in plant-plant interactions in wetland habitats based on a combination of flooding stress and soil texture. Later, the same authors demonstrated the additional negative impact of nutrient limitation [18]. Soil was reported to be a main driver in SGH applications along edapho-climatic gradients in semiarid environments [29], suggesting that there is also a possible key role of soil properties in other ecosystems, wetlands included. While edaphic and flooding gradients have been often studied, their possible interaction in shaping plant community responses was generally overlooked. 

We considered low- and middle-elevated salt marsh sites where the two key species *Limonium narbonense* (=*L. serotinum*, *L. vulgare* subsp. *serotinum*) and *Sarcornia fruticosa* (=*Salicornia fruticosa* and *Arthrocnemum fruticosum*) coexist with complementary abundance levels. In addition to the more general theoretical objective on SGH occurrence, this study also aimed to contribute to clarifying the interplay between these two species, in which one can be considered either the foremost competitor or the nurse of the community, and by which the magnitude and direction of the net interaction changes along the stress gradient. We hypothesised that controversies reported in literature could be explained by the interaction of the abiotic stress levels (i.e., flooding intensity) with soil properties (i.e., soil texture), which could amplify the stress, hence fostering the interaction shift detection.

## 2. Results

### 2.1. Intra- and Interspecific Competition Affect Plant Growth Traits

Plant growth traits showed large variability in both species and differed depending on the plant community, with more substantial changes related to *L. narbonense* traits (see Materials and Methods and Table 1).

**Figure 1:**
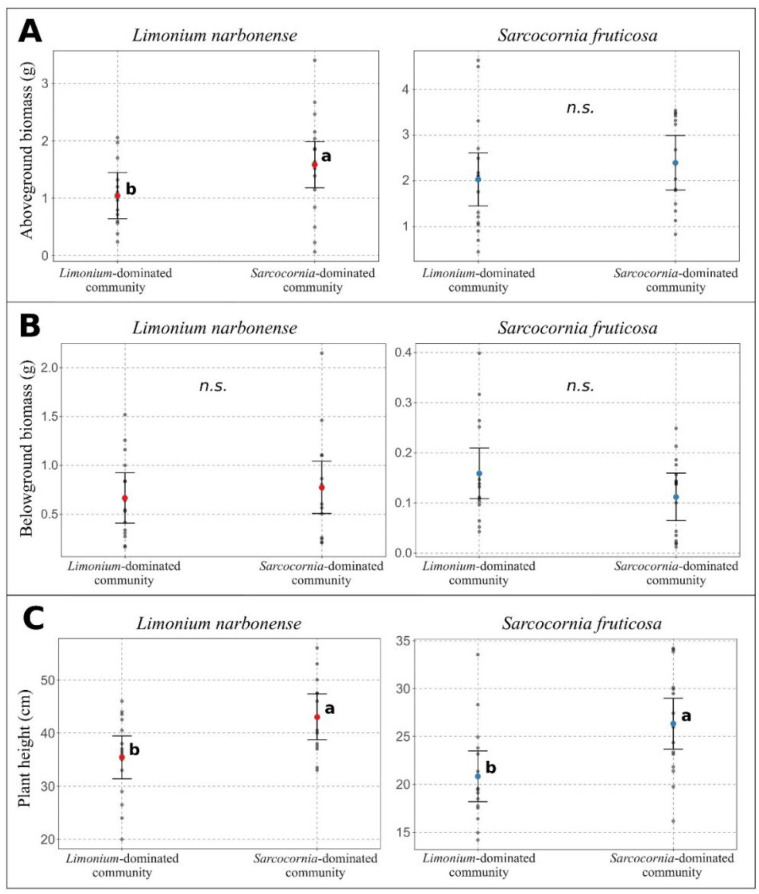
**Intra**- **and interspecific competition in *Limonium narbonense* and *Sarcocornia fruticosa*.** Competition was assessed on plant growth traits, i.e., above- (**A**) and belowground biomass (**B**) and plant height (**C**). Letters refer to statistical differences between traits of plant individuals growing in *Limonium*-dominated or *Sarcocornia*-dominated plant communities, based on Student’s *t*-test (*p* < 0.05). *n*.s. refers to statistically non-significant differences.

Aboveground biomass of *L. narbonense* and plant height of both species were significantly lower in *Limonium*-dominated plant communities compared to *Sarcocornia*-dominated communities (Figure 1A,C). A non-significant difference was recorded for the aboveground biomass of *S. fruticosa*. Moreover, the belowground biomass of both species was not affected by the type of plant community (Figure 1B).

### 2.2. Plant Community Does Not Influence Flooding Stress Responses

**Figure 2:**
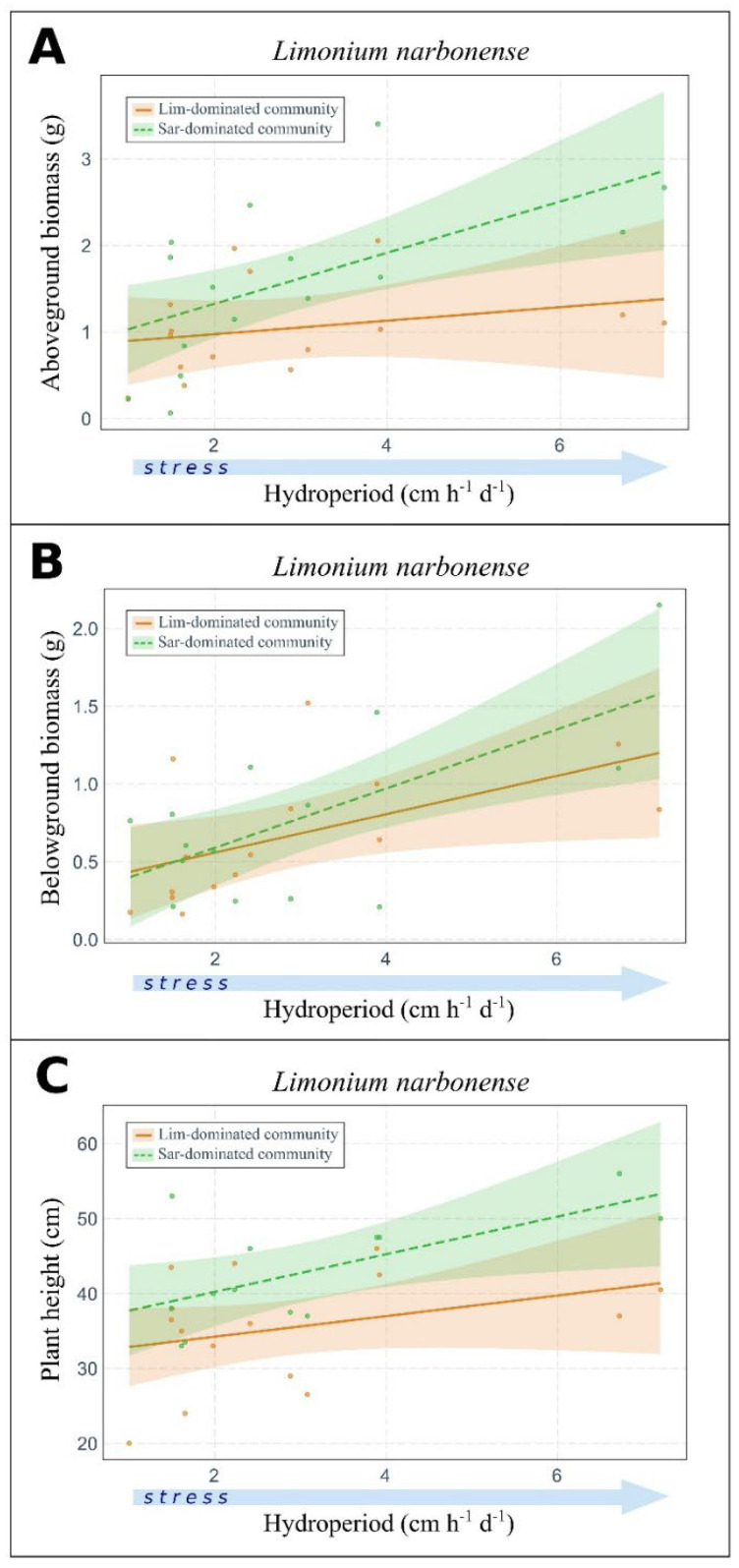
**Effect of flooding intensity on *Limonium narbonense* growth traits.** Changes of above- ((**A**) R^2^ = 0.35) and belowground biomass ((**B**), R^2^ = 0.37) and plant height ((**C**) R^2^ = 0.38) along the flooding gradient in monospecific stands or *Sarcocornia fruticose*-dominated plant communities. Light blue arrows indicate the increasing stress gradient of flooding. Only significant relationships obtained from ANOVA applied on linear models are shown (see Table S1). The interaction of flooding intensity and plant community type was not found to be statistically significant.

Flooding stress (i.e., flooding intensity) stimulated *L. narbonense* growth (Figure 2), with above- and belowground biomass and plant height increasing along the flooding-stress gradient. Differences were found in the response slopes of aboveground biomass and plant height between the two plant communities, but independently of their interaction with flooding intensity (Table S1).

*S. fruticosa* did not show any significant response in the traits studied along the flooding gradient, except for a significant difference in plant height between the plant community types (Table S1).

### 2.3. Soil Properties and Flooding Drive Plant Fitness Response

**Figure 3:**
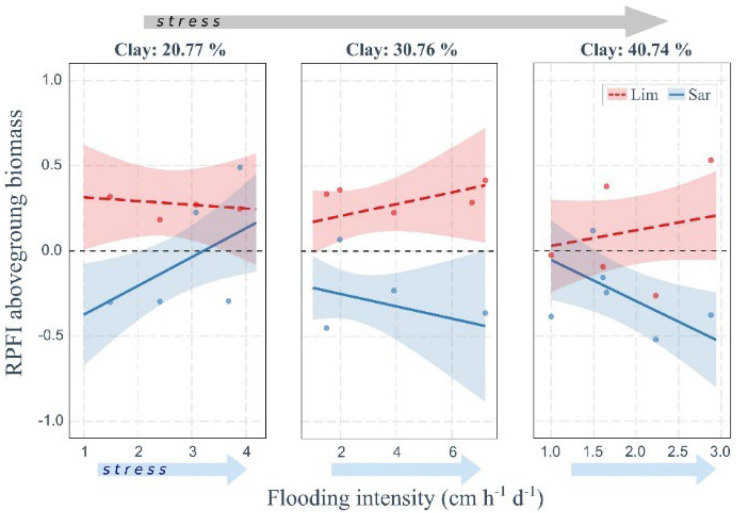
**Combined effect of flooding intensity and soil texture on plant fitness in terms of aboveground biomass.** Positive values of RPFI denote larger fitness of the species when growing in plurispecific stands compared to monospecific plant communities. Arrows indicate the increasing stress gradient of flooding (light blue arrow) or of harsher soil conditions (grey arrow, i.e., increasing soil clay content). Only significant relationships obtained from the ANOVA applied on linear models are shown (see Table S2, R^2^ = 0.56). Lim or Sar labels indicate the target species *Limonium narbonense* or *Sarcocornia fruticosa*, respectively. Significant differences in slopes at different levels of clay content in soil were found for *S. fruticosa* only (see Table S2).

**Figure 4:**
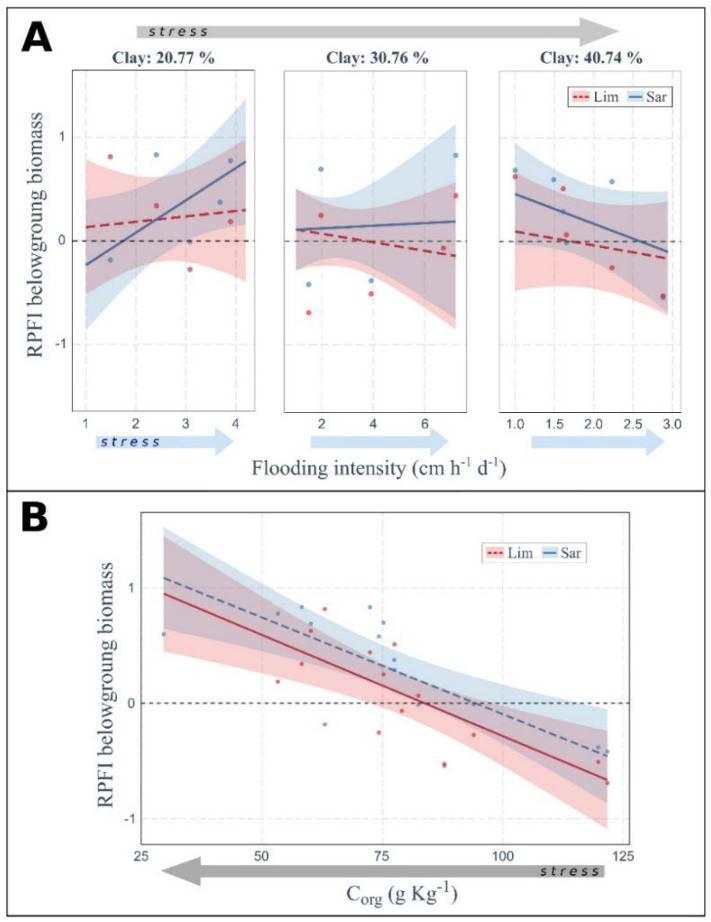
**Combined effects of flooding intensity and soil texture and of soil organic C on plant fitness in terms of belowground biomass.** Positive values of RPFI denote larger fitness of the species when growing in plurispecific stands compared to monospecific plant communities. Arrows indicate the increasing stress gradient of flooding (light blue arrow) or of harsher soil conditions (grey arrow, i.e., increasing soil clay content (**A**) or decreasing soil organic C (**B**). Only significant relationships obtained from the ANOVA applied on linear models are shown (see Table S3, R^2^ = 0.21 for (**A**); R^2^ = 0.59 for (**B**)). Lim or Sar labels indicate the target species *Limonium narbonense* or *Sarcocornia fruticosa*, respectively. Non-significant difference of RPFI between the two species was recorded.

**Figure 5:**
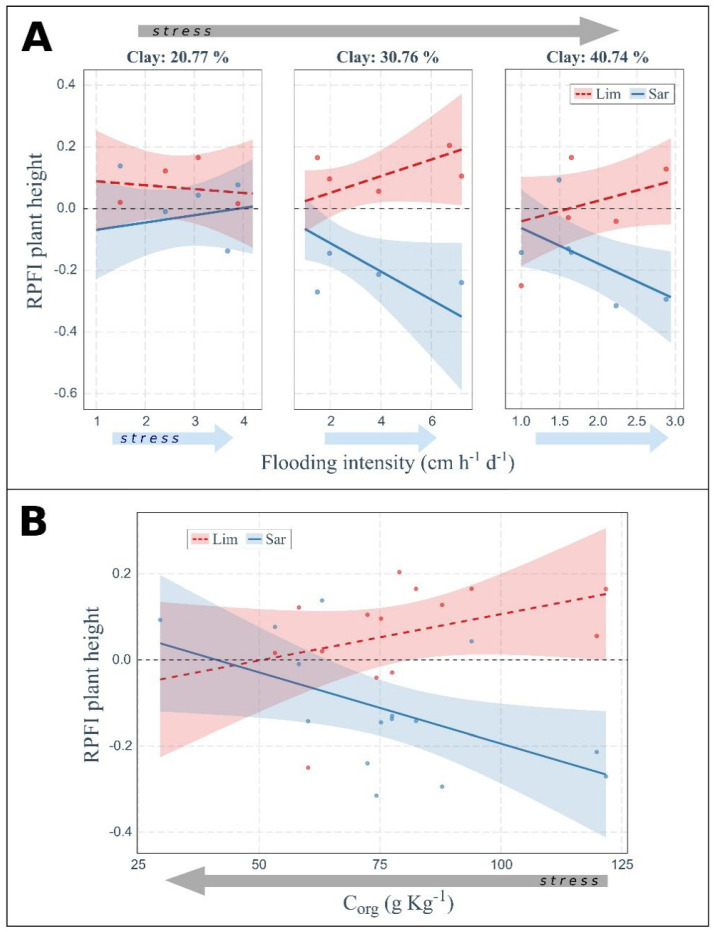
**Combined effects of flooding intensity and soil texture and of soil organic C on plant fitness in terms of plant height.** Positive values of RPFI denote larger fitness of the species when growing in plurispecific stands compared to monospecific plant communities. Arrows indicate the increasing stress gradient of flooding (light blue arrow) or of harsher soil conditions (grey arrow, i.e., increasing soil clay content (**A**) or decreasing soil organic C (**B**)). Only significant relationships obtained from the ANOVA applied on linear models are shown (see Table S4, R^2^ = 0.50 for (**A**); R^2^ = 0.52 for (**B**)). Lim or Sar labels indicate the target species *Limonium narbonense* or *Sarcocornia fruticosa*, respectively. Statistically significant differences of RPFI were found between the two species (both (**A**) and (**B**)). Significant differences in slopes were found for *S. fruticosa* only ((**A**), see Table S4).

Plant fitness, as assessed by the relative plant fitness index (RPFI), was affected not only by the increase of flooding intensity but also by soil properties (Figure 3, Figure 4 and Figure 5). In general, *L. narbonense* fitness was higher in *Sarcocornia*-dominated communities (RPFI > 0) while *S. fruticosa* showed the opposite trend (generally RPFI < 0). Moreover, plant fitness of both species changed with increasing stress resulting from the combination of flooding and edaphic stress, i.e., at increasing clay content of the soil and decreasing soil organic C.

The RPFI of the aboveground biomass of *L. narbonense* seemed almost independent of flooding and soil-texture gradients (Figure 3). On the other hand, RPFI of *S. fruticosa* tended to decrease with increasing flooding stress and clay content in soil, but with the remarkable exception at low clay content (left panel in Figure 3), where RPFI increased with increasing flooding intensity, with positive values indicating a higher fitness in mixed stands compared to monospecific communities. A similar result was also found for the RPFI of the belowground biomass (Figure 4A), where interspecific differences were less pronounced due to higher frequency of negative and positive RPFI values for *L. narbonense* and *S. fruticosa*, respectively. The RPFI response pattern for the two species was also confirmed when considering plant height (Figure 5A). In particular, at high clay content in soil (right panel in Figure 5A) and low flooding stress, both species showed RPFI values close to zero, indicating an almost null effect of the community type on species fitness. At increasing flooding stress, RPFI values progressively increased for *L. narbonense* and decreased for *S. fruticosa*, suggesting a better performance in mixed communities and in conspecific stands, respectively.

In addition to soil texture, organic C content in the soil affected plant fitness in terms of belowground biomass and plant height. The RPFI of belowground biomass of both species increased in soils with low organic C content regardless of the intensity of flooding stress (Figure 4B). Moreover, the RPFI of plant height of both species did not depend on flooding stress but increased in soils with low organic C in *S. fruticosa* while decreasing in *L. narbonense* (Figure 5B).

## 3. Discussion

This study aimed at assessing how the combination of flooding and edaphic stressors shapes plant-plant interactions in salt marshes. We hypothesised that soil properties can exacerbate the effect of flooding and consequently impact on net facilitative or competitive interactions. Indeed, our results showed that the relative plant fitness index (RPFI) increased with increasing stress, following the stress gradient hypothesis (SGH), but with a predominant role of soil properties in defining the stress gradients. Below, we discuss these novel findings in the context of existing knowledge and how our study revealed new insights into the concept of the SGH.

### 3.1. Effect of Plant-Plant Interactions on Plant Traits

*L. narbonense* generally showed a larger fitness, based on measured growth traits, in mixed-species stands compared to conspecific stands. This finding could be attributed to a predominant and negative effect of intraspecific competition as compared to intraspecific facilitation. This could be attributed to a net negative effect in conspecific stands or a prevailing positive effect of interspecific facilitation by *S. fruticosa* as compared to interspecific competition in mixed communities. Our data cannot directly separate the relative contributions of intra- and interspecific interactions to the observed pattern. However, evidence from previous studies could help in disentangling the possible mechanistic explanations. Indeed, a negative relationship between plant traits and population density had already been observed in *L. narbonense* [9] and in other wetland species forming dense monospecific stands [30], supporting the hypothesis of high levels of intraspecific competition under such conditions. Moreover, species of *Limonium* genus operate with both clonal and sexual reproduction [31] and their phenotypic plasticity towards abiotic stresses allow them to thrive in harsh environments, not suitable for other plants. Therefore, dense *Limonium*-dominated communities can result from a combination of different reproductive strategies and high plasticity, consistent with a large competition ability supported by our observations in monospecific stands. Moreover, the growth-form of basal rosettes in *L. narbonense* can lead to a larger cover compared to, e.g., the suffrutex form of *S. fruticosa*, where articles are thin and vertically oriented. Species with rosette growth form are considered to be weak competitors [32] compared to species showing higher canopy and larger biomass [32,33], consistent with our observations on *L. narbonense* in mixed-species stands.

A high competition potential of *L. narbonense* in dense, monospecific stands is also in agreement with the reduced growth of *S. fruticosa* in *Limonium*-dominated stands. Interestingly, such trends were recorded only in the cases of aboveground biomass and plant height (hence not for belowground biomass), suggesting that the competition potential of *L. narbonense* may rely on the capability for light interception. Indeed, *L. narbonense* has expanded leaves that can easily intercept the light much more than the vertical and slender green articles of *S. fruticosa*. Leaf area and leaf spatial distribution are key factors for light competition [34,35]. Plants with rosette growth form can react with hyponastic responses to flooding [36] and leaf plasticity in *L. narbonense* is also associated with changes in leaf area [9] and petiole elongation [36], further supporting its large competitiveness. Despite *S. fruticosa* appeared less competitive compared to *L. narbonense*, it is well-acclimated to flooding. It probably takes advantage of the reduced surface-area-to-volume ratio of the green photosynthetic shoots to decrease evapotranspiration [37], and of their succulence to increase plant tolerance to salinity, common traits in many wetland species [38,39].

### 3.2. Effect of Flooding on Plant Traits

Often, the study of plant-plant interaction in harsh environments, such as salt marshes, is complicated by the occurrence of multiple stressors, possibly interacting and producing non-additive, synergistic as well as antagonistic effects. Plant interspecific competition is known to vary along abiotic stress gradients, especially in wetlands and riparian systems [28,40]. In our work, flooding stress seemed to shape *L. narbonense* traits more than plant competition. On the contrary, *S. fruticosa* traits did not vary significantly along the flooding gradient, either in *Limonium*- or in *Sarcocornia*-dominated plant communities. Our findings are consistent with previous studies reporting a negative effect of flooding on leaf area of *L. narbonense* and shoot length of *S. fruticosa* [9], apparently unrelated to changes in plant biomass or height. Flooding is effective in segregating species richness of wetland communities [41] and despite a likely negative impact on plant biomass, recent studies report a negligible or even positive effect of flooding on other growth traits, depending on genotypic and phenotypic plasticity of the wetland plant [42,43]. Our findings suggest that flooding induces the production of small leaves and stems [9] counterbalanced by an increase in their number, compensating for the resulting loss in plant biomass. This was true for the flooding gradient considered, a substantial part of the whole elevation distribution of the two target species [44]. Flooding stress can accelerate leaf renewal, especially when flooding combines with other abiotic stresses e.g., salinity. Moreover, the physiological responses reported in literature for these two halophytes suggest the lack of growth limitation upon flooding stress [45].

The increase in plant height with increasing flooding stress is a common escape strategy of wetland plants for coping with submergence [46]. Both species are able to elongate when under water [47] and our data suggest that this acclimation trait is more pronounced in *L. narbonense*. Leaf elongation is highly species-specific [48] and represents a quick strategy to overcome short or incidental periods of submergence, by quickly restoring leaf contact with air and improving oxygen diffusion from shoots to roots [48,49]. Previous observations in laboratory-based studies have reported the ability of *S. fruticosa* to ‘snorkel’ by rapidly developing new and longer articles as a response to complete submergence [47]. In our field-based study, *S. fruticosa* height was not directly affected by flooding whereas petiole elongation in *L. narbonense* was highly dependent on the degree of flooding stress. It is possible that elongation in *S. fruticosa* is modulated by other additional factors that combine with the flooding stress e.g., phytotoxins developed in the anoxic flooded soil. This hypothesis can potentially explain why elongation was clearly boosted in laboratory-controlled conditions [47] but not observed in our field study.

The larger belowground biomass in *L. narbonense* upon flooding can probably be ascribed to the development of new adventitious roots, a common trait of flood-tolerant species to tackle soil anoxia [50], but not to an accumulation of carbon reserves in plant organs, which seems not to occur under abiotic stress [45]. Despite no data on root type being collected in this study (e.g., number or biomass of adventitious vs. seminal roots), the response observed for the two target species could provide indirect support to our explanatory hypothesis based on adventitious root development in *L. narbonense*. Indeed, *S. fruticosa* has surficial roots (usually less than 10 cm depth, [51]) providing access to oxygen at the upper soil layers [52]. In contrast, *L. narbonense* has a deeper vertical rhizome (up to 30–50 cm depth [53,54]) possibly requiring the development of numerous adventitious roots to support internal aeration. In fact, the root oxygen levels in *L. narbonense* are already dramatically reduced when partially submerged [47], increasing the demand for oxygen.

### 3.3. Effect of Flooding and Soil Texture on Plant Fitness

Based on our results, both plant-plant interactions and flooding stress could have an impact on the fitness of the two target halophytes. The use of the RPFI allowed us to jointly assess the effect of these two variables and to include additional potential stressors related to soil properties, depicting a more comprehensive overview of the interplay of the considered factors. In general, *L. narbonense* fitness was larger in mixed communities (RPFI > 0) and at increasing flooding stress. *S. fruticosa* showed, instead, a clear preference for conspecific communities (RPFI < 0) and exhibited a more pronounced effect of the interaction between flooding and soil texture.

The increase of *L. narbonense* fitness (aboveground biomass and plant height) with increasing flooding stress, could result from decreased competition with *S. fruticosa*, which is known to be less abundant and reduced in size in low elevated areas of salt marshes [44]. However, an increase in facilitation, with *S. fruticosa* acting as a nurse towards *L. narbonense*, could also have taken place. *Arthrocnemum subterminale*, a species closely related to *S. fruticosa*, was reported to facilitate other salt marsh species by locally decreasing soil salt levels and creating niches in elevation due to local sediment deposition [55]. The presence of *S. fruticosa* may therefore create beneficial niches for *L. narbonense*, in terms of a possible reduction of soil-related stressors (e.g., salt stress). Moreover, based on the contrasting root depth of the two species, with a shallow root system in *S. fruticosa* and a vertical rhizome that can reach more than 50 cm of depth in *L. narbonense* [54], the occurrence of a belowground interspecific competition might be excluded. Accordingly, *L. narbonense* in mixed stands could take advantage of its phenotypic plasticity in low elevated areas, and of the possible facilitation effect of *S. fruticosa*, in the absence of the intraspecific competition pressure occurring in conspecific communities (see above).

Contrary to *L. narbonense*, *S. fruticosa* fitness was larger in conspecific stands (RPFI < 0) but reduced in sandy soils at increasing flooding stress. Green articles of *S. fruticosa* can elongate when submerged for prolonged time [47]. However, sand content in salt marsh soils facilitates the drainage of flooded water during low tides [56] and reduces the exposure time of the plant to submergence. In these conditions, the species can probably re-allocate resources to increase its biomass [9] rather than elongating. This increase in biomass could drive a larger intraspecific competition and determine the decrease of plant fitness observed at increasing flooding stress in conspecific stands (RPFI ~ 0). Aboveground biomass and plant height were found to be larger in the closer species *Arthrocnemum macrostachyum* in the middle-upper elevations of salt marshes [57]. This suggests a larger intraspecific competition of suffrutex species at low flooding stress, consequently explaining the increase in *S. fruticosa* fitness recorded for clayey soils. 

A similar trend was observed for plant height, with *S. fruticosa* fitness increasing at high stress (high flooding and clay content in soil) in conspecific stands. Conditions of prolonged flooding, due to the compact clayey soil preventing drainage, force the species to elongate [47], thus possibly reducing intraspecific competition for space. In addition, intraspecific facilitation mechanisms could also be acting. Indeed, according to the SGH, the net effect of interactions results from the combination of competition and facilitation that act simultaneously [25,58]. Therefore, the above-discussed creation of niches in elevation and possible reduction in salt stress levels mediated by *S. fruticosa* could result in facilitation effects, supporting not only heterospecific beneficiaries (i.e., *L. narbonense* individuals) but also conspecific individuals exposed to stressful conditions.

The different niches occupied by the roots of the two target species can also explain the data recorded for the belowground biomass of *L. narbonense* along the flooding gradient, with RPFI values progressively shifting from positive to negative. It is possible that, in clayey soils and at increasing flooding stress, roots of *L. narbonense* compete for space with those of *S. fruticosa*. In fact, adventitious roots developing at the rhizome-shoot junction of *L. narbonense* are possibly restricted by the surficial root system of *S. fruticosa* [51]. Moreover, root aeration in *L. narbonense* is less efficient during submergence compared to *S. fruticosa* [47]. Therefore, the reduction in fitness of the root system of *L. narbonense* could be attributed to the combined restriction in tissue aeration and larger competition in heterospecific plant communities subject to abiotic stress (flooding or a combination of flooding and edaphic stress).

*S. fruticosa* fitness based on belowground biomass showed the same trend but in most cases with positive values of RPFI. The higher plant fitness in plurispecific communities could be due to niche partitioning as also proposed for *L. narbonense* (see above). The increase of intraspecific competition of *S. fruticosa* is even more pronounced at increasing flooding stress and in sandy soils. Nevertheless, competition/facilitation processes almost disappeared in soils with high clay content (RPFI ~0), especially at larger flooding stress. This level of abiotic stress is probably severe for both species. Soil is completely anoxic and competition/facilitation is known to be ineffective at critical stress levels [59,60]. Roots of halophytes are exposed to phytotoxin intrusion that challenges plant survival [61], and phytotoxicity can therefore be considered as an additional stressor able to neutralise possible facilitation/competition interactions.

### 3.4. Effect of Flooding and Soil Organic C on Plant Fitness

In both species, the effect of soil organic C on plant fitness of both species was significant in terms of belowground biomass and plant height, but not of aboveground biomass. Moreover, this effect was larger compared to the consequences of flooding stress, driving *L. narbonense* and *S. fruticosa* fitness regardless to the flooding intensity.

Organic C limitation produced higher fitness, in terms of belowground biomass, for both species in plurispecific communities. Based on the niche theory, niche construction by a species can either generate interspecific facilitation or strengthen interspecific competition [62]. In our study-case, a reduction in competition based on niche partitioning was expected, considering the different growth forms of the two species with root systems exploring different soil layers. Consequently, competition for nutrient uptake could be indirectly reduced and plant fitness promoted [24]. However, the occurrence of facilitative mechanisms acting by increasing stress (organic C limitation) should not be excluded. Both species can contribute to the soil organic C and nutrient pools that are usually very low in these salt marsh soils, due to the high content of carbonates and sand restricting soil cation-exchange capacity [63,64]. At larger organic C in soil, the process seemed reversed, probably due to the larger growth of plants in C-rich soils and the larger interspecific competition for resources. This explains the core idea of the SGH, for which facilitative interactions shift to competition as abiotic stress conditions ameliorate [16]. Here, the conspecific association of stands seemed the best strategy for both plant species to increase their fitness. 

Plant fitness based on height showed contrasting trends between the two species, with higher RPFI values for *L. narbonense* in plurispecific stands and for *S. fruticosa* in conspecific communities, with a tendency for competition/facilitation to produce a substantially null effect (RPFI ~ 0) at low flooding intensity and at limiting soil organic C.

The low-oxygen escape strategy determines leaf/shoot elongation in plants subject to flooding [36]. By elongating, the plant immediately benefits from ‘snorkelling’ and from oxygen diffusion from shoots to roots thanks to the aerenchyma tissue [49]. *S. fruticosa* seems to facilitate this trait in individuals of the same species as well as in *L. narbonense*. The beneficial stressors boosting shoot/leaf elongation could possibly be attributed to the growth form of *S. fruticosa* and to its larger contribution of lignin to the soil organic pool. In fact, the shrub-like growth form of *S. fruticosa* can reduce light penetration through the canopy [9]. The flooding stress, combined with the reduced light availability, can consequently boost shoot/leaf elongation. In addition, *S. fruticosa* could locally increase the organic C stock in the soil, due to the larger contribution in lignin compared to *L. narbonense* (about 13% in *S. fruticosa* and 8% in *L. narbonense* [65,66]), and hence increase plant fitness of *L. narbonense* and *S. fruticosa* individuals in heterospecific and conspecific communities, respectively. On the other hand, at limiting C levels in soil, possible facilitation mechanisms can be negligible because of the harsh conditions that both species experience.

## 4. Conclusions

Our work stressed some critical aspects on the application of the stress gradient hypothesis (SGH) in harsh environments, specifically in salt marshes and wetlands.

Following the SGH, the increase in facilitation proceeds in parallel to a decrease in competition, resulting in a stress-related response, where net effects are magnified at intermediate stress levels. In our study, this was not always the case, because of the concomitant occurrence of edaphic stresses, producing a synergistic magnification of the primary effect of flooding as in the case of soil texture interactive effects, or prevailing on it as in the case of soil organic C content. In agreement with the SGH expectations, the response pattern along the explored multivariate stress gradient of both target species was consistent with a net positive plant-plant interaction effect (i.e., a net increase of facilitative interactions and/or a net decrease of competitive interactions) at increasing flooding stress and intermediate edaphic stress (clay content in soil). However, the monotone or hump-shaped severity-interaction relationship described in the SGH was not observed along the flooding-stress gradient or the combined flooding-edaphic stress gradients explored. Interestingly, our data highlight that net plant-plant interactions do not respond univocally to any stressor, but rather respond with specific shape only to specific types of abiotic stress or to specific combinations of different stressors. Moreover, high levels of the abiotic stress do not necessarily correspond to a net increase in facilitation (or a net decrease in competition). The intensity of the stress can be critical for the growth of a plant, challenging its survival at a level where plant-plant interaction effects are negligible, such as at very low C content in soil. 

In conclusion, our results stressed the importance of considering context-dependent features, such as edaphic conditions in our study-case, as additional abiotic stresses in SGH applications, as well as the need to include them in the SGH formula e.g., by using a normalised index summarising the main stressors of the target environment. Moreover, each stressor should be weighted in order to consider the selection of a narrower range of the stress gradient and correct the application of the SGH approach on specific field situations. Complementary studies could further explain the evidence highlighted by the SGH approach and unravel the contribution of competition or facilitation as well as of plant-, soil- or microbiome-mediated drivers of plant-plant interactions.

## 5. Materials and Methods

### 5.1. Study Area and Sampling Design

The Marano and Grado Lagoons (centroid coordinates: 45°42′50″ N, 13°20′30″ E, Northern Adriatic Sea) cover an area of about 160 km^2^. These are among the largest transitional coastal ecosystems of the Mediterranean Sea and both are part of the European Natura 2000 network (SAC/SPA IT3320037). The lagoons are delimited by six barrier islands and most of the emerged surface is covered by salt marshes fragmented by a complex network of channels and tidal flats. The average tidal fluctuation and the spring tidal range are 65 and 105 cm, respectively [67].

Two key salt marsh species were selected according to their frequency and abundance in the studied salt marshes, namely *Limonium narbonense* (= *L. serotinum*, *L. vulgare* subsp. *serotinum*) and *Sarcocornia fruticosa* (= *Salicornia fruticosa*, *Arthrocnemum fruticosum*). Both species are perennial, showing a similar distribution along the major range of the studied flooding gradient. *L. narbonense* (family Plumbaginaceae) is a rhizomatous geophyte with basal leaves (rosette) and bare flowering stems. *S. fruticosa* (family Amaranthaceae) is a leafless suffrutex, with green succulent articles (swollen twigs) supported by woody basal stems with branched shallow roots.

Flooding stress highly depends on site elevation, mainly affecting tidal flooding timing and depth. Soil texture further exacerbates flooding duration by means of a faster (coarse particles) or slower (fine particle) drainage of the water during low tides. In this light, the following two salt marshes were chosen for this study representing a relevant gradient in elevation and soil texture. Belvedere salt marsh has mainly a loamy soil texture and it is located in the inner part of the lagoon, while Marina di Macia is a back-barrier salt marsh with predominantly sandy soils.

**Figure 6:**
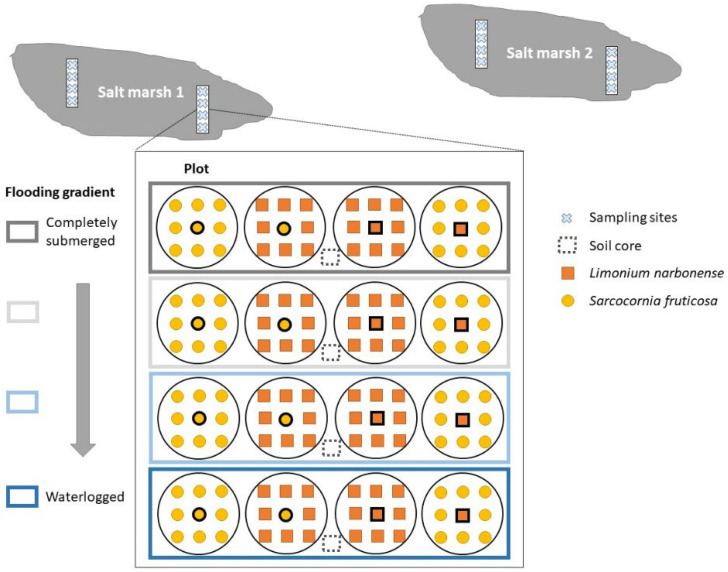
**Schematic view of the sampling design.** Four transects were selected in two salt marshes following the flooding gradient. Intra- and interspecific competition of the salt marsh species *Limonium narbonense* and *Sarcocornia fruticosa* were assessed in circular plots of 20 cm diameter. The central individual in each plot was sampled for further analyses. In each sampling site, the flooding intensity was monitored for 14 days and a core of surficial soil was collected.

The sampling design was aimed at disentangling intra- and interspecific competition along the study stress gradients (flooding alone or combined with edaphic stressors). Therefore, two elevation transects were positioned in each salt marsh (four transects in total), and four sampling sites were selected in each transect (along the elevation gradient) as a proxy for flooding intensity. Four circular plots of 20 cm diameter (area of 1256 cm^2^) were established (4 × 4 × 4 = 64 plots) at each sampling site (Figure 6). Each plot was characterised by one target species (species A) surrounded by a monospecific plant community dominated by the same species (species A) or by the second selected species (species B). Within each plot, the target species positioned at the centre of the plot was collected and further processed (see below). In each transect, about 100 g of superficial (about 10 cm depth) soil was also collected for further analysis. Sampling was carried out during the late growing season (July–September).

### 5.2. Flooding Stress

Tidal flooding affects the sampling sites differently based on the site elevation and the distance of the salt marshes from the back-barrier islands delimiting the Marano and Grado lagoons. The flooding stress intensity was measured at each plot using data loggers (HOBO U-20L-01, Onset). The sensors were positioned at the ground level, with a logging interval of 5 min along a complete tide cycle (2 weeks, including a shift from spring tide to neap tide). Pressure data, recorded with the loggers, were automatically converted to water level using the HOBOware^®^Pro software (version 3.7.14, Onset Corporation) and normalised per hour per day. The flooding intensity ranged from 1 to 7.2 cm h^−^^1^ d^−^^1^ (Table 1).

### 5.3. Plant Traits and Soil Analyses

Plant height of the target species (individual at the centre of each plot) was recorded. Dry mass of above- and belowground biomass was measured on a balance after 72 h of desiccation at 70 °C.

The soil samples were homogenised and then used for further analyses. Soil texture was measured in a Bouyoucos’ cylinder with an ASTM 152H hydrometer. Organic C content was measured using a CHNS Elemental Analyzer (Vario Microcube, Elementar, Langenselbold, Germany) after acidifying to remove the inorganic fraction of C from the soil samples. 

Data collected on plant traits and soil properties are briefly summarised in Table 1.

### 5.4. Data Analyses

The effect of abiotic stresses (flooding and edaphic factors) on plant traits and plan-plant interactions were analysed. Intra- and interspecific interactions (i.e., competition and facilitation) of the two selected species were investigated through the use of the relative plant fitness index (RPFI), a modified index based on the relative interaction index reported by [68]. RPFI was calculated using by following Equation (1):

(1) $$RPFI=\frac{TraitAB-TraitAA}{TraitAB+TraitAA}$$

where *TraitAB* is the plant trait measured in the species A when present in a monospecific plant community dominated by the species B, and *TraitAA* is the same trait measured in species A when present in a monospecific plant community dominated by the same species A. The index varies from −1 to +1. Negative values denote larger fitness of the target species (Species A) in conspecific plant communities (dominated by the same species A), while positive values denote a larger fitness in mixed plant communities (in our sampling design, dominated by species B). When the index is 0, positive (facilitation) and negative (competition) interaction among species are counterbalanced. 

Student’s *t*-test was applied to evaluate differences in plant traits related to intra- or interspecific competition (i.e., of plants growing in monospecific communities composed of the same species or by the second selected species) in the two selected species, after verifying the assumptions of data normality (Shapiro-Wilk test > 0.05) and homoscedasticity (Bartlett’s test > 0.05).

Linear models (LMs) were applied to examine the effect of flooding alone or in combination with edaphic factors on plant traits and on the RPFI. The salt marsh was considered as random factor. LMs were applied using the “nlme” R package [69]. Model assumptions were verified using diagnostic plots and Shapiro-Wilk normality test (*p* > 0.05) on model residuals; the models met linear model assumptions.

Slopes of significant interactions between two independent variables (i.e., flooding and edaphic factors) were further analysed using the “emtrends” function within the “emmeans” package [70].

Significant models were plotted using the “ggplot” package. All graphs and statistical analyses were performed in R statistical software (version 3.6.0, R Core Team, Vienna, Austria) [71].

## Figures and Tables

**Table 1:** **Plasticity of plant traits and variability of edaphic factors within the sampling sites.** The range (minimum and maximum) and the mean ± standard deviation is reported for plant traits of *Limonium narbonense* and *Sarcocornia fruticosa* and for soil texture and organic C content. *N* refers to the number of replicates.

	Minimum	Maximum	*N*	Mean ± SD
***Limonium narbonense***				
Plant height (cm)	12.0	56.0	30	38.0 ± 9.6
Aboveground biomass (g)	0.06	3.40	29	1.31 ± 0.80
Belowground biomass (g)	0.16	2.15	29	0.72 ± 0.48
***Sarcocornia fruticosa***				
Plant height (cm)	14.2	34.2	32	23.6 ± 5.8
Aboveground biomass (g)	0.45	4.63	32	2.21 ± 1.12
Belowground biomass (g)	0.01	0.40	32	0.13 ± 0.09
**Flooding intensity (cm h^−1^ d^−1^)**	1.0	7.2	16	2.9 ± 1.8
**Soil texture**				
Clay (%)	11	51	16	31.4 ± 10.2
Silt (%)	5	31	16	16.3 ± 8.4
Sand (%)	34	83	16	52.3 ± 14.4
**Soil organic C (%)**	3.0	12.2	16	7.7 ± 2.3

## Data Availability

Data is contained within the article and Supplementary Materials. Additional data is available on request from the corresponding author.

## Supplementary Materials

The following supporting information can be downloaded at: https://www.mdpi.com/article/10.3390/plants11151940/s1. Table S1. ANOVA results applied on linear models testing the effect of hydroperiod and plant community on key traits of Limonium narbonense and Sarcocornia fruticosa. Table S2. ANOVA results applied on linear models testing the effect of species, hydroperiod and soil properties on RPFI calculated on aboveground biomass. Table S3. ANOVA results applied on linear models testing the effect of species, hydroperiod and soil properties on RPFI calculated on belowground biomass. Table S4. ANOVA results applied on linear models testing the effect of species, hydroperiod and soil properties on RPFI calculated on plant height.

## References

[1] Grime J.P. (1979). Plant Strategies and Vegetation Processes.

[2] Pezeshki S.R., DeLaune R.D. (2012). Soil oxidation-reduction in wetlands and its impact on plant functioning. Biology.

[3] Davy A.J., Brown M.J.H., Mossman H.L., Grant A. (2011). Colonization of a newly developing salt marsh: Disentangling independent effects of elevation and redox potential on halophytes. J. Ecol..

[4] Ponnamperuma F.N., Kozlowski T. (1984). Effects of Flooding on Soils. Flooding and Plant Growth.

[5] Colmer T.D. (2003). Long-distance transport of gases in plants: A perspective on internal aeration and radial oxygen loss from roots. Plant Cell Environ..

[6] Pedersen O., Colmer T., Sand-Jensen K. (2013). Underwater photosynthesis of submerged plants-recent advances and methods. Front. Plant Sci..

[7] Winkel A., Colmer T.D., Pedersen O. (2011). Leaf gas films of Spartina anglica enhance rhizome and root oxygen during tidal submergence. Plant Cell Environ..

[8] Brooker R.W., Maestre F.T., Callaway R.M., Lortie C.L., Cavieres L.A., Kunstler G., Liancourt P., Tielbörger K., Travis J.M.J., Anthelme F. (2008). Facilitation in plant communities: The past, the present, and the future. J. Ecol..

[9] Pellegrini E., Boscutti F., De Nobili M., Casolo V. (2018). Plant traits shape the effects of tidal flooding on soil and plant communities in saltmarshes. Plant Ecol.

[10] Bertness M.D., Callaway R. (1994). Positive interactions in communities. Trends Ecol. Evol..

[11] Brooker R.W., Scott D., Palmer S.C.F., Swaine E. (2006). Transient facilitative effects of heather on scots pine along a grazing disturbance gradient in Scottish moorland. J. Ecol..

[12] Bertness M.D., Gough L., Shumway S.W. (1992). Salt tolerances and the distribution of fugitive salt marsh plants. Ecology.

[13] Irving A.D., Bertness M.D. (2009). Trait-dependent modification of facilitation on cobble beaches. Ecology.

[14] Fogel B.N., Crain C.M., Bertness M.D. (2004). Community level engineering effects of Triglochin maritima (*Seaside arrowgrass*) in a salt marsh in northern New England, USA. J. Ecol..

[15] Levine J.M., Hacker S.D., Harley C.D.G., Bertness M.D. (1998). nitrogen effects on an interaction chain in a salt marsh community. Oecologia.

[16] Maestre F.T., Callaway R.M., Valladares F., Lortie C.J. (2009). Refining the stress-gradient hypothesis for competition and facilitation in plant communities. J. Ecol..

[17] Bonanomi G., Stinca A., Chirico G.B., Ciaschetti G., Saracino A., Incerti G. (2016). Cushion plant morphology controls biogenic capability and facilitation effects of Silene acaulis along an elevation gradient. Funct. Ecol..

[18] Huckle J.M., Marrs R.H., Potter J.A. (2002). Interspecific and intraspecific interactions between salt marsh plants: Integrating the effects of environmental factors and density on plant performance. Oikos.

[19] García-Cervigón A.I., Gazol A., Sanz V., Camarero J.J., Olano J.M. (2013). Intraspecific competition replaces interspecific facilitation as abiotic stress decreases: The shifting nature of plant–plant interactions. Perspect. Plant Ecol. Evol. Syst..

[20] Callaway R.M., Brooker R.W., Choler P., Kikvidze Z., Lortie C.J., Michalet R., Paolini L., Pugnaire F.I., Newingham B., Aschehoug E.T. (2002). Positive interactions among alpine plants increase with stress. Nature.

[21] Daleo P., Iribarne O. (2009). Beyond Competition: The stress-gradient hypothesis tested in plant-herbivore interactions. Ecology.

[22] Maestre F.T., Cortina J. (2004). Do positive interactions increase with abiotic stress? A test from a semi-arid steppe. Proc. R. Soc. Lond. Ser. B Biol. Sci..

[23] Barchuk A.H., Valiente-Banuet A., Díaz M.P. (2005). Effect of shrubs and seasonal variability of rainfall on the establishment of Aspidosperma quebracho-blanco in two edaphically contrasting environments. Austral Ecol..

[24] Zhang L., Shao H. (2013). Direct plant-plant facilitation in coastal wetlands: A review. Estuar. Coast. Shelf Sci..

[25] Malkinson D., Tielbörger K. (2010). What does the stress-gradient hypothesis predict? resolving the discrepancies. Oikos.

[26] Patel K.F., Fansler S.J., Campbell T.P., Bond-Lamberty B., Smith A.P., RoyChowdhury T., McCue L.A., Varga T., Bailey V.L. (2021). Soil texture and environmental conditions influence the biogeochemical responses of soils to drought and flooding. Commun. Earth Environ..

[27] Vittori Antisari L., Ferronato C., Pellegrini E., Boscutti F., Casolo V., de Nobili M., Vianello G. (2017). Soil properties and plant community relationship in a saltmarsh of the Grado and Marano Lagoon (northern Italy). J. Soils Sediments.

[28] Huckle J.M., Potter J.A., Marrs R.H. (2000). Influence of environmental factors on the growth and interactions between salt marsh plants: Effects of salinity, sediment and waterlogging. J. Ecol..

[29] Lima T.R.A., Martins F.R., Menezes B.S., Marquitti F.M.D., Sfair J.C., Silveira A.P., Araújo F.S. (2022). The stress gradient hypothesis explains plant-plant interaction networks in edapho climatic gradients. Acta Oecol..

[30] Cronk J.K., Fennessy M.S. (2001). Wetland Plants: Biology and Ecology.

[31] Róis A.S., Rodríguez López C.M., Cortinhas A., Erben M., Espírito-Santo D., Wilkinson M.J., Caperta A.D. (2013). Epigenetic rather than genetic factors may explain phenotypic divergence between coastal populations of diploid and tetraploid *Limonium* spp. (*Plumbaginaceae*) in Portugal. BMC Plant Biol..

[32] Dietz H., Steinlein T., Ullmann I. (1998). The role of growth form and correlated traits in competitive ranking of six perennial ruderal plant species grown in unbalanced mixtures. Acta Oecol..

[33] Keddy P., Fraser L.H., Wisheu I.C. (1998). A Comparative approach to examine competitive response of 48 wetland plant species. J. Veg. Sci..

[34] Black J.N. (1960). The significance of petiole length, leaf area, and light interception in competition between strains of subterrranean clover (*Trifolium subterraneum* L.) grown in swards. Aust. J. Agric. Res..

[35] Wang C., Liu J., Xiao H., Zhou J. (2016). Differences in leaf functional traits between Rhus typhina and native species. CLEAN-Soil Air Water.

[36] Bailey-Serres J., Voesenek L.A.C.J. (2008). Flooding stress: Acclimations and genetic diversity. Annu. Rev. Plant Biol..

[37] Mauseth J.D. (2000). Theoretical aspects of surface-to-volume ratios and water-storage capacities of succulent shoots. Am. J. Bot..

[38] Pan Y., Cieraad E., van Bodegom P.M. (2019). Are ecophysiological adaptive traits decoupled from leaf economics traits in wetlands?. Funct. Ecol..

[39] Flowers T.J., Colmer T.D. (2008). Salinity tolerance in halophytes. New Phytol..

[40] Chen Y.-H., Sun X.-S., Cui Y., Zhuo N., Wei G.-W., Luo F.-L., Zhang M.-X. (2021). Interacting Flooding and Competition Negatively Affect Growth of Riparian Species Dominating a Reservoir Shoreline. Water.

[41] Casanova M.T., Brock M.A. (2000). How do depth, duration and frequency of flooding influence the establishment of wetland plant communities?. Plant Ecol..

[42] Reents S., Mueller P., Tang H., Jensen K., Nolte S. (2021). Plant genotype determines biomass response to flooding frequency in tidal wetlands. Biogeosciences.

[43] Lan Z., Chen Y., Shen R., Cai Y., Luo H., Jin B., Chen J. (2021). Effects of flooding duration on wetland plant biomass: The importance of soil nutrients and season. Freshw. Biol..

[44] Silvestri S., Defina A., Marani M. (2005). Tidal regime, salinity and salt marsh plant zonation. Estuar. Coast. Shelf Sci..

[45] Pellegrini E., Forlani G., Boscutti F., Casolo V. (2020). Evidence of non-structural carbohydrates-mediated response to flooding and salinity in Limonium narbonense and Salicornia fruticosa. Aquat. Bot..

[46] Lou Y., Pan Y., Gao C., Jiang M., Lu X., Xu Y.J. (2016). Response of plant height, species richness and aboveground biomass to flooding gradient along vegetation zones in floodplain wetlands, northeast China. PLoS ONE.

[47] Pellegrini E., Konnerup D., Winkel A., Casolo V., Pedersen O. (2017). Contrasting oxygen dynamics in Limonium narbonense and Sarcocornia fruticosa during partial and complete submergence. Funct. Plant Biol..

[48] Herzog M., Pedersen O. (2014). Partial versus complete submergence: Snorkelling aids root aeration in Rumex palustris but not in R. acetosa. Plant Cell Environ..

[49] Colmer T.D., Flowers T.J. (2008). Flooding tolerance in halophytes. New Phytol..

[50] Pedersen O., Sauter M., Colmer T.D., Nakazono M. (2021). Regulation of root adaptive anatomical and morphological traits during low soil oxygen. New Phytol..

[51] Figueroa M.E., Castillo J.M., Redondo S., Luque T., Castellanos E.M., Nieva F.J., Luque C.J., Rubio-Casal A.E., Davy A.J. (2003). Facilitated invasion by hybridization of Sarcocornia species in a salt-marsh succession. J. Ecol..

[52] Zehnder A.J.B., Stumm W. (1988). Geochemistry and biogeochemistry of anaerobic habitats. Biology of Anaerobic Microorganisms.

[53] Pellegrini E., Petranich E., Acquavita A., Canário J., Emili A., Covelli S. (2017). Mercury uptake by halophytes in response to a long-term contamination in coastal wetland salt marshes (Northern Adriatic Sea). Environ. Geochem. Health.

[54] Boorman L.A. (1967). *Limonium vulgare* Mill. and *L. humile* Mill. J. Ecol..

[55] Callaway R.M. (1994). Facilitative and interfering effects of Arthrocnemum subterminale on winter annuals. Ecology.

[56] Bradley P.M., Morris J.T. (1990). Physical characteristics of salt marsh sediments: Ecological implications. Mar. Ecol. Prog. Ser..

[57] Gul B., Khan M.A. (1999). Effect of intraspecific competition and inundation regime on the growth of Arthrocnemum macrostachyum in a coastal swamp in Karachi, Pakistan. Pak. J. Bot..

[58] Callaway R.M., Walker L.R. (1997). Competition and facilitation: A synthetic approach to interactions in plant communities. Ecology.

[59] Choler P., Michalet R., Callaway R.M. (2001). Facilitation and competition on gradients in alpine plant communities. Ecology.

[60] Soliveres S., Eldridge D.J., Maestre F.T., Bowker M.A., Tighe M., Escudero A. (2011). Microhabitat Amelioration and reduced competition among understorey plants as drivers of facilitation across environmental gradients: Towards a unifying framework. Perspect. Plant Ecol. Evol. Syst..

[61] Lamers L., Govers L., Janssen I., Geurts J., Van der Welle M., Van Katwijk M., Van der Heide T., Roelofs J., Smolders A. (2013). Sulfide as a soil phytotoxin—a review. Front. Plant Sci..

[62] Kylafis G., Loreau M. (2011). Niche construction in the light of niche theory. Ecol. Lett..

[63] Vittori Antisari L., De Nobili M., Ferronato C., Natale M., Pellegrini E., Vianello G. (2016). Hydromorphic to subaqueous soils transitions in the central Grado lagoon (northern Adriatic Sea, Italy). Estuar. Coast. Shelf Sci..

[64] Pellegrini E., Contin M., Livia V.A., Chiara F., Nobili M.D. (2019). Soil organic carbon and carbonates are binding phases for simultaneously extractable metals (SEM) in calcareous saltmarsh soils. Environ. Toxicol. Chem..

[65] Carrasco-Barea L., Llorens L., Romaní A.M., Gispert M., Verdaguer D. (2022). Litter decomposition of three halophytes in a Mediterranean salt marsh: Relevance of litter quality, microbial activity and microhabitat. Sci. Total Environ..

[66] Buth G.J.C., Voesenek L.A.C.J., Huiskes A.H.L., Blom C.W.P.M., Rozema J. (1987). Decomposition of standing and fallen litter of halophytes in a Dutch salt marsh. Vegetation between Land and Sea: Structure and Processes.

[67] Ret M. (2006). Bilancio Idrologico e Circolazione Idrica della Laguna di Marano e Grado. Master’s Thesis.

[68] Armas C., Ordiales R., Pugnaire F.I. (2004). Measuring plant interactions: A new comparative index. Ecology.

[69] Pinheiro J. Nlme: Linear and Nonlinear Mixed Effects Models. R Package Version 3.1-98. http://cran.r-project.org/package=nlme.

[70] Russell L. Emmeans: Estimated Marginal Means, aka Least-Squares Means, R Package Version 2018, 1. https://CRAN.R-project.org/package=emmeans.

[71] R Core Team R (2020). A Language and Environment for Statistical Computing.

