# Supplementary material for: Flooding and Soil Properties Control Plant Intra- and Interspecific Interactions in Salt Marshes
Source: Plants (Basel). 2022 Jul 26;11(15):1940. doi: 10.3390/plants11151940 (PMC9331060; doi:10.3390/plants11151940)
Supplement: Supplementary file 1 [file plants-11-01940-s001.zip › plants-1808080-supplementary.pdf]

Supplementary Materials

Table S1. ANOVA results applied on linear models (Df = degrees of freedom, F value = F statistic, P = p value) testing the effect of hydroperiod and plant community on key traits of *Limonium narbonense* and *Sarcocornia fruticosa*, i.e. above- and belowground biomass and plant height. *** for $P < 0.001$, ** for $P < 0.01$, * for $P < 0.05$, • for $P < 0.07$.

	Df	F value	*P*	
**RPFI Aboveground biomass ~ Hydroperiod x Plant community**				
Limonium narbonense				
Hydroperiod	1	7.76	0.009	**
Plant community	1	4.93	0.035	*
Hydroperiod:plant community	1	2.63	0.117	
Residuals	26			
Sarcocornia fruticosa				
Hydroperiod	1	0.15	0.70	
Plant community	1	0.77	0.39	
Hydroperiod:plant community	1	0.15	0.70	
Residuals	27			
**RPFI Belowground biomass ~ Hydroperiod x Plant community**				
Limonium narbonense				
Hydroperiod	1	15.1	<0.001	**
Plant community	1	0.40	0.533	
Hydroperiod:plant community	1	0.69	0.415	
Residuals	25			
Sarcocornia fruticosa				
Hydroperiod	1	0.08	0.777	
Plant community	1	1.87	0.183	
Hydroperiod:plant community	1	0.21	0.648	
Residuals	26			
**RPFI Plant height ~ Hydroperiod x Plant community**				
Limonium narbonense				
Hydroperiod	1	8.20	0.009	**
Plant community	1	7.47	0.012	*
Hydroperiod:plant community	1	0.63	0.434	
Residuals	24			
Sarcocornia fruticosa				
Hydroperiod	1	0.09	0.762	
Plant community	1	8.46	0.007	**
Hydroperiod:plant community	1	0.34	0.567	
Residuals	28			

Table S2. ANOVA results applied on linear models (Df = degrees of freedom, F value = F statistic, *P* = p value) testing the effect of species (*Limonium narbonense* and *Sarcocornia fruticosa*), hydroperiod and soil properties (clay or organic C) on RPFI calculated on aboveground biomass. Differences in slopes were further analysed using the *emtrends* function. *** for $P < 0.001$, ** for $P < 0.01$, * for $P < 0.05$, • for $P < 0.07$.

	Df	F value	*P*	
**RPFI Aboveground biomass ~ Hydroperiod x Clay x Species**				
Hydroperiod	1	2.15	0.16	
Clay	1	1.84	0.19	
Species	1	22.0	<0.001	***
Hydroperiod:clay	1	2.36	0.14	
Hydroperiod:species	1	0.57	0.46	
Clay:species	1	0.09	0.76	
Hydroperiod:clay:species	1	6.47	0.02	*
Residuals	21			
Emtrends - Limonium narbonense	Df	estimate	P	
20.77-30.76	21	-0.06	0.73	
20.77-40.74	21	-0.11	0.73	
30.76-40.74	21	-0.06	0.73	
Emtrends - Sarcocornia fruticosa				
20.77-30.76	21	0.07	0.03	•
20.77-40.74	21	0.14	0.03	•
30.76-40.74	21	0.07	0.03	•

Table S3. ANOVA results applied on linear models (Df = degrees of freedom, F value = F statistic, *P* = p value) testing the effect of species (*Limonium narbonense* and *Sarcocornia fruticosa*), hydroperiod and soil properties (clay or organic C) on RPFI calculated on belowground biomass. Differences in slopes were further analysed using the *emtrends* function. *** for $P < 0.001$, ** for $P < 0.01$, * for $P < 0.05$, • for $P < 0.07$.

	Df	F value	*P*	
**RPFI Belowground biomass ~ Hydroperiod x Clay x Species**				
Hydroperiod	1	0.01	0.91	
Clay	1	0.12	0.73	
Species	1	1.42	0.25	
Hydroperiod:clay	1	3.90	0.06	•
Hydroperiod:species	1	0.33	0.57	
Clay:species	1	0.59	0.45	
Hydroperiod:clay:species	1	0.91	0.35	
Residuals	21			
**RPFI Belowground biomass ~ Hydroperiod x Corg x Species**				
Hydroperiod	1	0.03	0.86	
Corg	1	33.8	<0.001	***
Species	1	1.71	0.20	
Hydroperiod:Corg	1	0.14	0.71	
Hydroperiod:species	1	2.31	0.14	
Corg:species	1	0.16	0.69	
Hydroperiod:Corg:species	1	1.33	0.26	
Residuals	21			

Table S4. ANOVA results applied on linear models (Df = degrees of freedom, F value = F statistic, *P* = p value) testing the effect of species (*Limonium narbonense* and *Sarcocornia fruticosa*), hydroperiod and soil properties (clay or organic C) on RPFI calculated on plant height. Differences in slopes were further analysed using the *emtrends* function. *** for $P < 0.001$, ** for $P < 0.01$, * for $P < 0.05$, • for $P < 0.07$.

	Df	F value	*P*	
**RPFI Plant height ~ Hydroperiod x Clay x Species**				
Hydroperiod	1	0.73	0.40	
Clay	1	3.43	0.08	
Species	1	14.9	<0.001	***
Hydroperiod:clay	1	0.18	0.67	
Hydroperiod:species	1	3.52	0.07	•
Clay:species	1	0.96	0.34	
Hydroperiod:clay:species	1	3.94	0.06	•
Residuals	21			
Emtrends Lim	Df	estimate	*P*	
20.77-30.76	21	-0.04	0.33	
20.77-40.74	21	-0.08	0.33	
30.76-40.74	21	-0.04	0.33	
Emtrends Sar				
20.77-30.76	21	0.07	0.08	•
20.77-40.74	21	0.14	0.08	•
30.76-40.74	21	0.07	0.08	•
**RPFI Plant height ~ Hydroperiod x Corg x Species**				
Hydroperiod	1	0.77	0.39	
Corg	1	0.75	0.40	
Species	1	16.5	<0.001	***
Hydroperiod:Corg	1	0.003	0.95	
Hydroperiod:species	1	2.48	0.13	
Corg:species	1	8.29	0.009	**
Hydroperiod:Corg:species	1	1.34	0.26	
Residuals	21			
Emtrends Corg				
Lim-Sar	21	0.055	0.019	*
